# Acquired canalicular cryptorchidism mimicking testicular torsion following neonatal herniotomy: a rare cause of acute paediatric groin pain

**DOI:** 10.1093/jscr/rjaf960

**Published:** 2025-12-04

**Authors:** Christopher Bell, Cheng Ming Li, Raunakbir Bhatia, Robert George

**Affiliations:** Department of General Surgery, Broken Hill Base Hospital, 176 Thomas Street, Broken Hill 2880, Australia; Department of General Surgery, Broken Hill Base Hospital, 176 Thomas Street, Broken Hill 2880, Australia; Department of General Surgery, Broken Hill Base Hospital, 176 Thomas Street, Broken Hill 2880, Australia; Department of General Surgery, Broken Hill Base Hospital, 176 Thomas Street, Broken Hill 2880, Australia

**Keywords:** cryptorchidism, incarcerated testis, testicular torsion, paediatric surgery, inguinal herniotomy, rural surgery

## Abstract

We report a rare case of an 11-year-old boy presenting to a rural Australian hospital with acute left groin pain, a history of extreme prematurity, and bilateral inguinal herniotomies in infancy. Examination revealed a firm, tender, irreducible mass in the left inguinal canal and an empty left hemiscrotum. The right testis was retractile and non-tender. Doppler ultrasound showed absent blood flow to the left testis, prompting urgent surgical exploration. Intraoperatively, the testis was incarcerated within the left inguinal canal amid dense post-surgical adhesions. After adhesiolysis, the testis was viable and mobilized for tension-free orchidopexy via a subdartos pouch. The contralateral testis was also fixated. We hypothesize acquired canalicular cryptorchidism due to post-herniorrhaphy scarring causing vascular compromise from extrinsic compression. This rare mimic of torsion underscores the importance of recognizing atypical presentations of a threatened testis and demonstrates timely surgery is feasible in rural settings with remote specialist support.

## Introduction

Cryptorchidism is common in preterm infants and often coexists with inguinal hernias due to shared embryological pathways [1]. Though typically congenital, acquired testicular ascent is increasingly recognized, with prior inguinal herniotomy a known risk factor [1, 2]. Undescended testes are more susceptible to torsion, and their impalpable position may delay recognition and intervention [3].

We present a rare case of intra-canalicular testicular incarceration mimicking torsion in a child with prior herniotomy. This case underscores the importance of early recognition of uncommon presentations of a threatened testis and demonstrates how paediatric scrotal emergencies can be safely managed in rural settings with appropriate specialist oversight.

## Case report

An 11-year-old boy presented to a rural Australian emergency department with 12 h of worsening left groin pain. His parents noted recent comments about ‘having only one testicle’, though no prior concern had been raised. There was no history of trauma, gastrointestinal or urinary symptoms. He was born at 25 weeks’ gestation and had undergone bilateral inguinal herniotomies in infancy, without documented orchidopexy. His history also included craniofacial abnormalities requiring surgery and a diagnosis of autism spectrum disorder. He was otherwise well and attending school.

**Figure 1:**
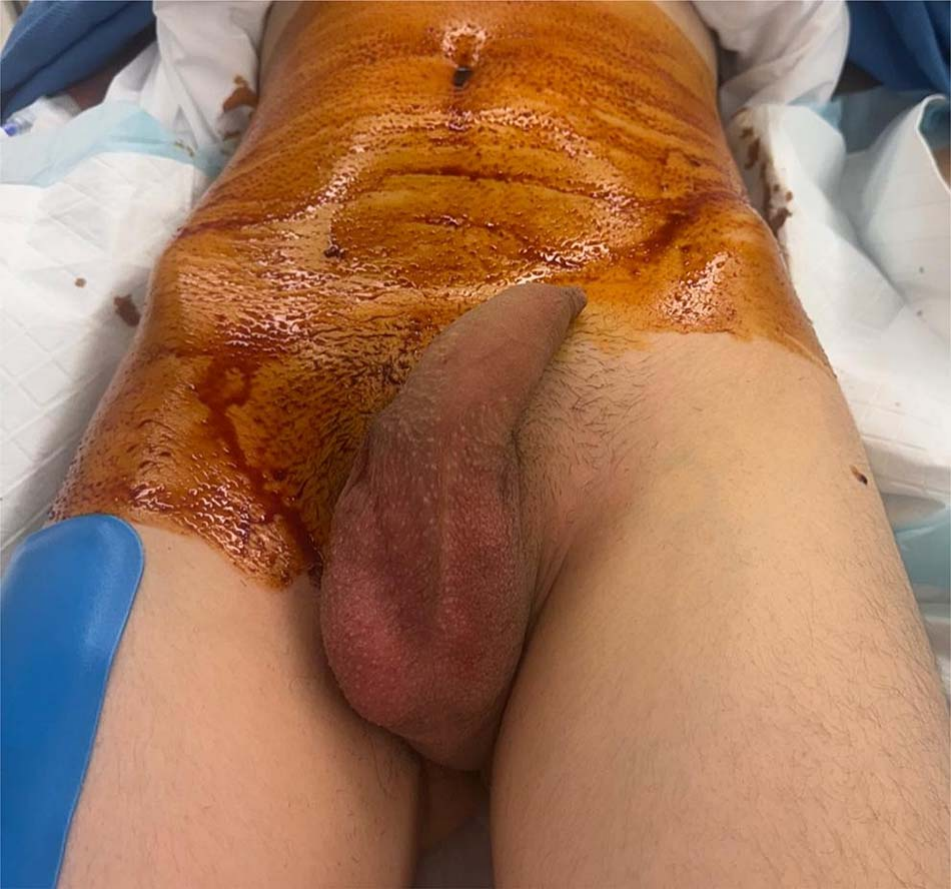
Pre-operative image showing left groin swelling and empty left hemiscrotum following anaesthetic induction.

On examination, the right testis was retractile but non-tender and easily reducible into the scrotum. The left hemiscrotum was empty. There was a tender, firm, immobile mass palpable in the left inguinal region just lateral to the pubic tubercle (Fig. 1). There was no overlying erythema. Attempts at reduction with analgesia and Valsalva were unsuccessful.

**Figure 2:**
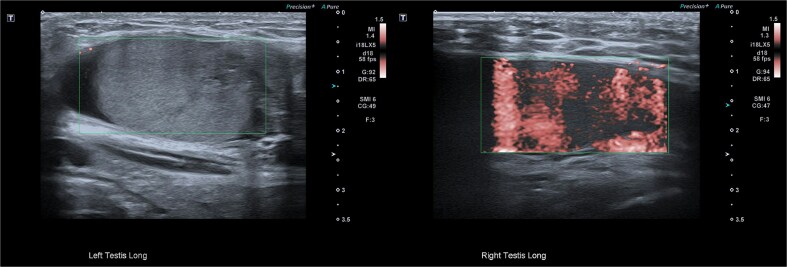
Pre-operative ultrasonographic image demonstrating left and right intracanalicular testis. The left testis has absent colour Doppler flow and heterogeneous echotexture concerning for compromised perfusion.

Although testicular torsion is a clinical diagnosis, we felt that the complexity of this case warranted ultrasound, which was immediately available and demonstrated no vascular flow to the left testis and normal perfusion to the right (Fig. 2). Given these findings and suspicion of torsion, emergency exploration was undertaken at our rural facility under paediatric surgical advice obtained from a tertiary centre.

**Figure 3:**
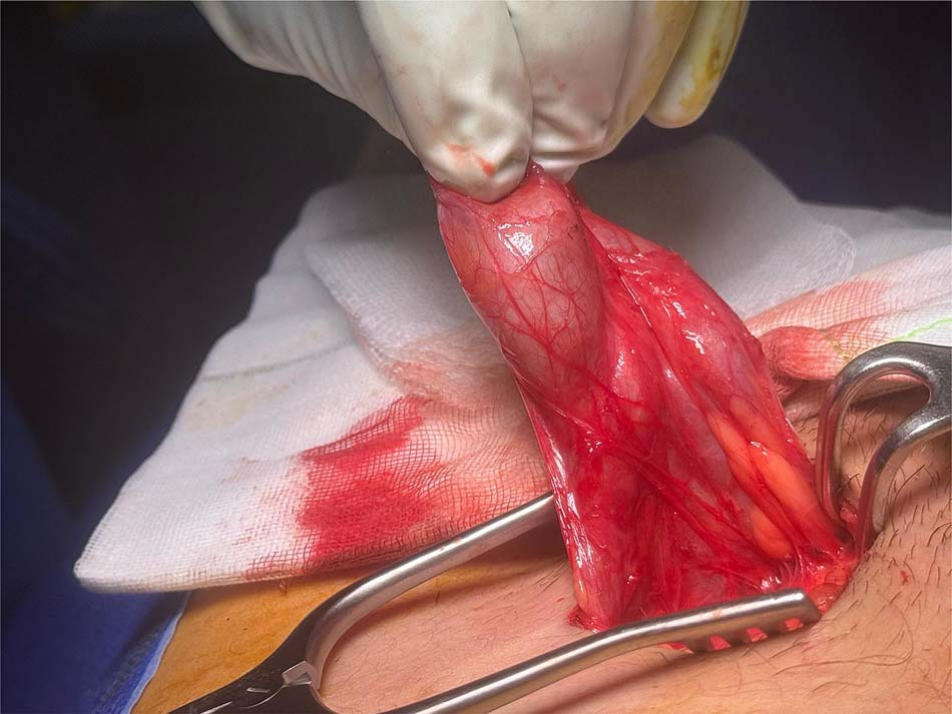
Intraoperative view of the left testis incarcerated within the inguinal canal and surrounded by adhesions.

Following general anaesthetic induction, manual reduction into the scrotum was once again attempted but was unsuccessful. Exploration of the inguinal canal was planned to assess testicular viability, degree of impaction and adhesions, and the length of testicular pedicle. A left inguinal incision was made, and dissection was carried down to the inguinal canal where the left testis was found incarcerated by dense fibrotic adhesions (Fig. 3).

**Figure 4:**
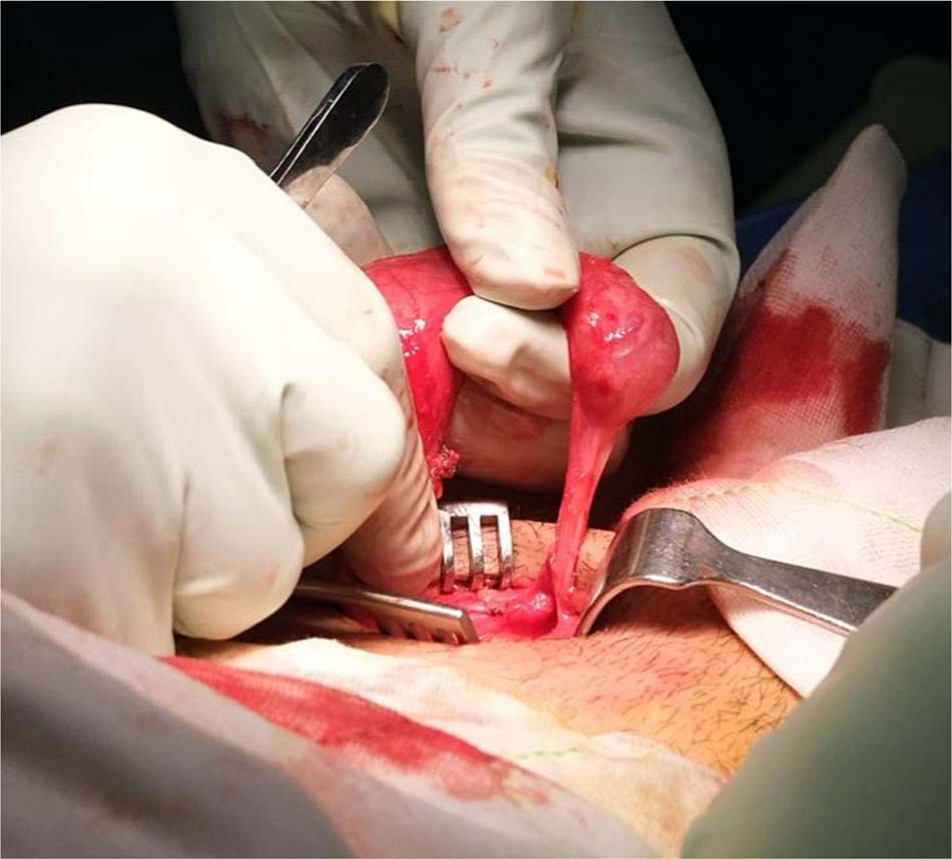
Left testis following adhesiolysis, demonstrating an elongated gubernaculum tethered high in the scrotum.

**Figure 5:**
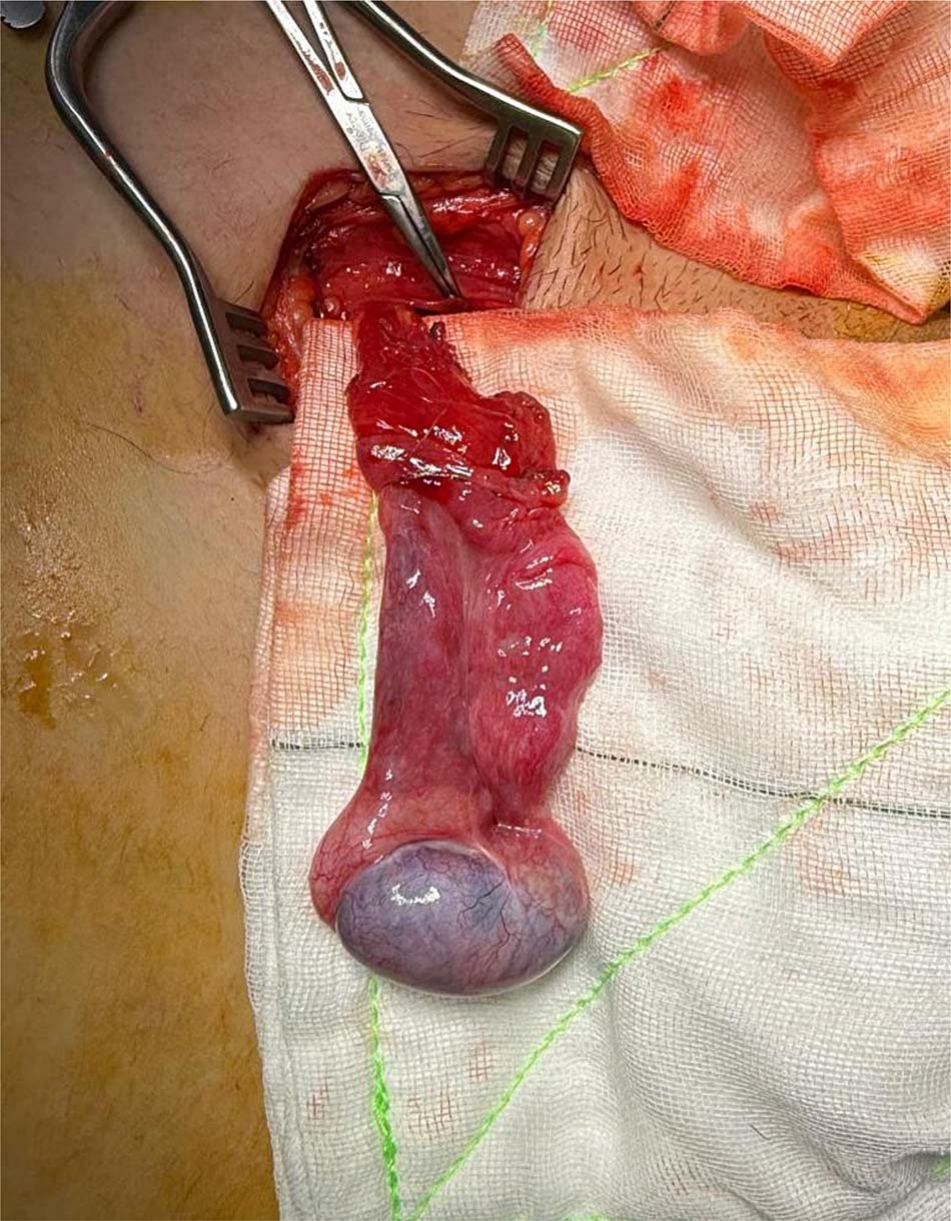
Completely mobilized left testis, showing viable appearance and adequate cord length for tension-free mobilization.

**Figure 6:**
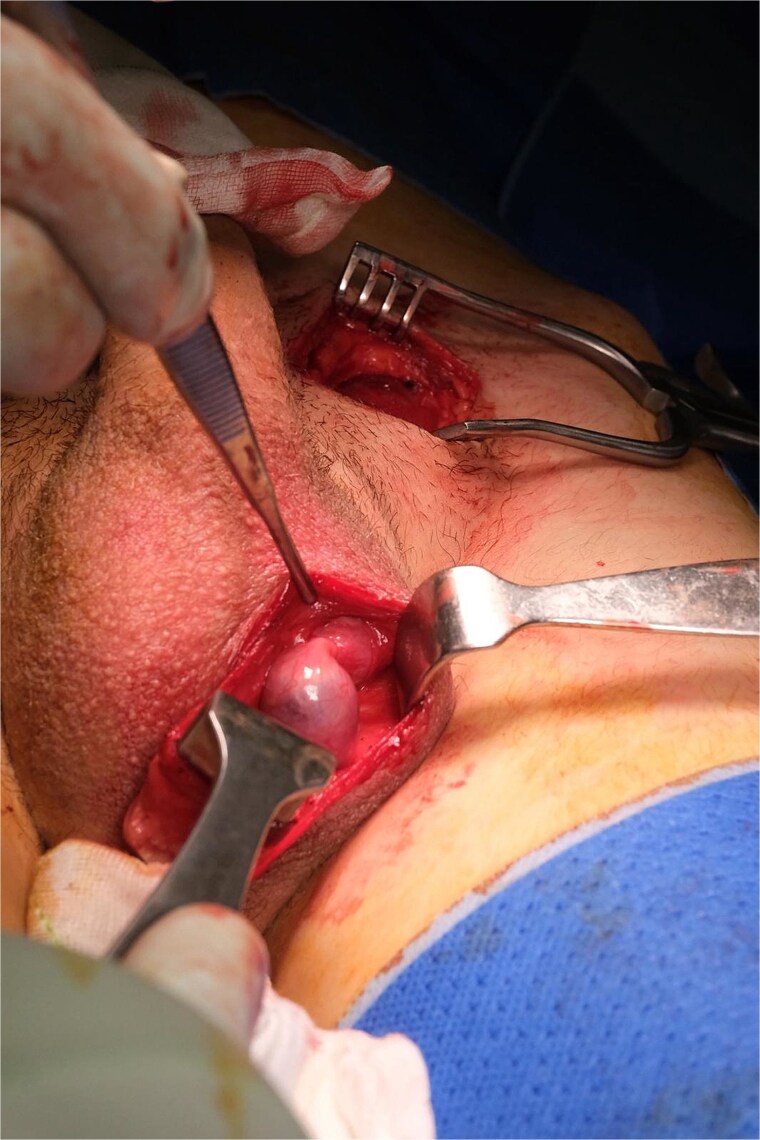
Creation of subdartos pouch in the left hemiscrotum prior to testicular fixation.

An elongated gubernaculum tethered high in the scrotum was also noted (Fig. 4). Following careful dissection, the testis was freed, revealing a viable appearance and adequate cord length for scrotal mobilization (Fig. 5). A subdartos pouch was created via a left paramedian scrotal incision, and the testis was delivered into the scrotum via a tunnel created by gentle blunt dissection (Fig. 6). The testis was fixated in three-points with a 4-0 polydioxanone suture. The contralateral hemiscrotum was also explored utilizing the same incision, where the right testis was found to have a normal lie and attachments. It was likewise fixated.

**Figure 7:**
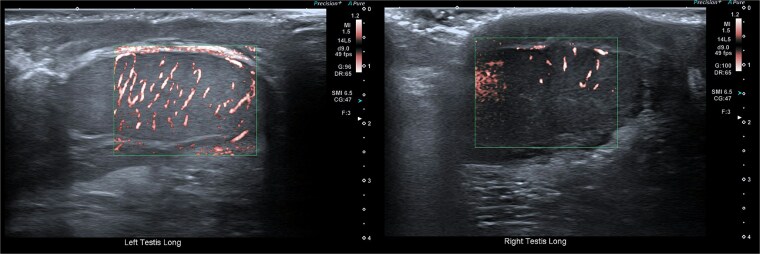
Follow-up ultrasonographic image at 4 weeks post-operatively, demonstrating the left testis within the scrotum, with restored homogeneous echotexture and normal intratesticular vascularity on colour Doppler.

Closure was performed in layers, and local anaesthetic was infiltrated. The procedure was well tolerated. Follow-up at four weeks showed preserved testicular position with normal Doppler inflow and normal echotexture (Fig. 7).

## Discussion

Acquired cryptorchidism, or testicular ascent, refers to a testis previously documented in the scrotum that later becomes undescended. Though historically under-recognized, it is now considered more common than congenital cryptorchidism, particularly in pre-pubertal boys [1]. Proposed mechanisms include persistent fibrous remnants of the processus vaginalis, inelasticity of the spermatic cord, and scarring from prior inguinal surgery [1, 2]. Risk factors include retractile testis, prior inguinal surgery, prematurity, and abnormal gubernacular attachments [1].

Torsion is a recognized complication of ectopic testis. Individuals with congenital cryptorchidism are reported to have as much as a ten-fold increased risk [3]. While this association is less directly studied in ascending or acquired cryptorchidism, similar anatomic factors—such as lack of scrotal fixation and underlying abnormal attachments—may likewise predispose to torsion events [1, 2]. Importantly, clinical recognition is often delayed, with salvage rates significantly lower than in scrotal torsion [3].

Prompt surgical exploration remains the gold standard in any child with suspected torsion. Colour Doppler ultrasonography (CDUS) is the imaging modality of choice in equivocal situations, approaching 95% sensitivity and 95% specificity in experienced hands [4]. However, in early or intermittent torsion, arterial flow may be preserved, leading to false-negative studies [5]. Imaging of ectopic testis is further challenged by anatomic inaccessibility, limited acoustic windows, and obscured Doppler signals [6]. In our case, rapid bedside CDUS demonstrated absent perfusion and reinforced the decision to explore. As imaging was immediately available and did not delay theatre access, its use was justified. However, in less clear-cut or delayed scenarios, exploration should proceed regardless of imaging findings [7].

Notably in our patient, no torsion was apparent, and the testis was viable. Instead, it appeared that post-herniorrhaphy scarring had led to intra-canalicular entrapment, resulting in progressive vascular compromise from extrinsic compression—either on the testicular pedicle or the testis itself. Ascent was likely permitted by an abnormally elongated and highly fixated gubernaculum. Partial torsion with spontaneous detorsion remains a plausible alternative, although the fixed position of the testis argued against significant rotational mobility. To our knowledge, no similar case has been described in the literature.

While rare, this presentation underscores the importance of maintaining a high index of suspicion for testicular compromise in children with atypical groin findings. Early recognition, timely operative decision-making, and appropriate remote specialist input enabled definitive management. Recent Australian data confirm that, with adequate support, testicular salvage rates in rural centres are comparable to urban care, with no significant difference in orchidectomy rates despite increased travel distances or lower socioeconomic status [8]. This aligns with current Royal Australasian College of Surgeons guidelines endorsing local exploration when interhospital transfer may delay care [7], reinforcing that safe and timely management of paediatric scrotal emergencies is achievable in rural settings.
